# Neuronal deletion of GSK3β increases microtubule speed in the growth cone and enhances axon regeneration via CRMP-2 and independently of MAP1B and CLASP2

**DOI:** 10.1186/1741-7007-12-47

**Published:** 2014-06-12

**Authors:** Márcia A Liz, Fernando M Mar, Telma E Santos, Helena I Pimentel, Ana M Marques, Marlene M Morgado, Sílvia Vieira, Vera F Sousa, Hayley Pemble, Torsten Wittmann, Calum Sutherland, James R Woodgett, Mónica M Sousa

**Affiliations:** 1 Nerve Regeneration Group, IBMC - Instituto de Biologia Molecular e Celular, 4150-180 Porto, Portugal; 2 Instituto de Ciências Biomédicas Abel Salazar – ICBAS, 4050-313 Porto, Portugal; 3 Department of Cell and Tissue Biology, University of California, San Francisco, CA 94145, USA; 4 Diabetic and Cardiovascular Medicine, University of Dundee, Ninewells Hospital, Dundee DD1 9SY, UK; 5 Samuel Lunenfeld Research Institute, Mount Sinai Hospital, University of Toronto, Toronto, ON M5G 1X5, Canada

**Keywords:** GSK3β, CRMP-2, Growth cone, Microtubule, Axon regeneration

## Abstract

**Background:**

In the adult central nervous system, axonal regeneration is abortive. Regulators of microtubule dynamics have emerged as attractive targets to promote axonal growth following injury as microtubule organization is pivotal for growth cone formation. In this study, we used conditioned neurons with high regenerative capacity to further dissect cytoskeletal mechanisms that might be involved in the gain of intrinsic axon growth capacity.

**Results:**

Following a phospho-site broad signaling pathway screen, we found that in conditioned neurons with high regenerative capacity, decreased glycogen synthase kinase 3β (GSK3β) activity and increased microtubule growth speed in the growth cone were present. To investigate the importance of GSK3β regulation during axonal regeneration *in vivo*, we used three genetic mouse models with high, intermediate or no GSK3β activity in neurons. Following spinal cord injury, reduced GSK3β levels or complete neuronal deletion of GSK3β led to increased growth cone microtubule growth speed and promoted axon regeneration. While several microtubule-interacting proteins are GSK3β substrates, phospho-mimetic collapsin response mediator protein 2 (T/D-CRMP-2) was sufficient to decrease microtubule growth speed and neurite outgrowth of conditioned neurons and of GSK3β-depleted neurons, prevailing over the effect of decreased levels of phosphorylated microtubule-associated protein 1B (MAP1B) and through a mechanism unrelated to decreased levels of phosphorylated cytoplasmic linker associated protein 2 (CLASP2). In addition, phospho-resistant T/A-CRMP-2 counteracted the inhibitory myelin effect on neurite growth, further supporting the GSK3β-CRMP-2 relevance during axon regeneration.

**Conclusions:**

Our work shows that increased microtubule growth speed in the growth cone is present in conditions of increased axonal growth, and is achieved following inactivation of the GSK3β-CRMP-2 pathway, enhancing axon regeneration through the glial scar. In this context, our results support that a precise control of microtubule dynamics, specifically in the growth cone, is required to optimize axon regrowth.

## Background

In the central nervous system (CNS), axons mostly fail to regenerate given the inhibitory environment and the lack of activation of neuronal-intrinsic regeneration-associated pathways. It is, however, possible to stimulate the intrinsic growth capacity of CNS axons. When the peripheral branch of a dorsal root ganglia (DRG) neuron is injured prior to lesion to its central branch (conditioning lesion), the central axons can overcome the glial scar inhibitory effect and regenerate [1]. Although several molecules have been identified as required for the conditioning effect, none was proven to be sufficient and necessary to mimic the conditioning lesion, probably as a combination of various mechanisms is needed.

Microtubule organization is crucial for axon regeneration. While CNS axons respond to injury by forming a retraction bulb with a disorganized network of microtubules, peripheral nervous system (PNS) axons form a growth cone composed by a backbone of stable microtubules and dynamic microtubules in the axon tip [2]. Pharmacological destabilization of microtubules converts a growth cone into a retraction bulb, and stabilization leads to the formation of growth cone-like structures [2] and increases axonal regeneration *in vivo*, after spinal cord injury (SCI) [3]. Accordingly, histone deacetylase 6 (HDAC6) inhibition enhances tubulin acetylation and promotes axon growth on inhibitory substrates [4]. In contrast, it has been shown that in the PNS, axons have a higher regenerative capacity as they activate a program leading to the activation of histone deacetylase 5 (HDAC5) and to a lower microtubule stability close to the injury site [5].

Here, we further explored the mechanisms underlying the gain of regenerative capacity in conditioned DRG neurons and identified glycogen synthase kinase 3β (GSK3β) as an important player in this process. GSK3β is a serine/threonine kinase that phosphorylates several microtubule-interacting proteins, namely collapsin response mediator protein 2 (CRMP-2), microtubule-associated proteins (MAP1B and tau), microtubule plus end-tracking proteins (adenomatous polyposis coli and cytoplasmic linker associated protein 2 (CLASP2)) and the microtubule-depolymerizing factor stathmin [6]. The activity of GSK3β is regulated by phosphorylation. Under resting conditions, the kinase is constitutively active following phosphorylation of its Tyr216 residue, located in the kinase domain [7]. The mechanism of Tyr216 phosphorylation has been proposed to correspond to an autocatalytic event [8] although in neurons, kinases such as Pyk2 and Fyn have also been proposed as responsible for this phosphorylation [9,10]. Exposure of neuroblastoma cells to the general tyrosine phosphatase inhibitor ortho-vanadate led to increased levels of Tyr216 phosphorylation, raising the hypothesis that a group of yet unidentified tyrosine phosphatases might regulate the phosphorylation of Tyr216 [11]. Nevertheless, regulation of GSK3β activity is generally seen as resulting from inactivation by phosphorylation of its Ser9 residue [12]. In response to extracellular signals that activate the phosphatidylinositide 3-kinase (PI3K) pathway, protein kinase B (AKT) is activated triggering the inactivation of GSK3β by phosphorylation of Ser9 [13].

The array of GSK3β substrates suggests that it is a key kinase regulating microtubule dynamics and an attractive molecule to study in the context of axon growth. However, controversy exists regarding GSK3β modulation during axonal regeneration as pharmacological inhibition of GSK3β was shown to promote either axonal growth [14] or myelin-dependent axon outgrowth inhibition [15]. While most data relating GSK3β and axonal regeneration relied on semi-specific pharmacological inhibitors tested *in vitro*, to further determine the relevance of GSK3β during axonal regeneration *in vivo*, we used three GSK3β genetic mouse models with different levels of kinase activity: constitutively active GSK3βSer9Ala knockin (KI) mice [16], GSK3β knockout heterozygous mice [17] and mice with neuron-specific deletion of GSK3β. Our data show that increased microtubule dynamics in the growth cone are achieved following inactivation of the GSK3β-CRMP-2 pathway, enhancing axon regeneration through the glial scar.

## Results

### Decreased GSK3β activity through downregulation of P-Tyr216 is related to increased axonal regeneration

**Figure 1:**
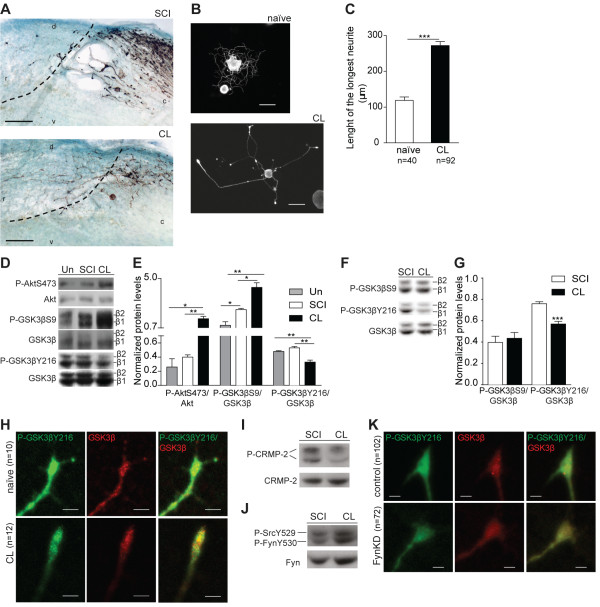
**Decreased GSK3β activity through downregulation of P-Tyr216 is related to increased axonal growth. (A)** Representative images of dorsal column fibers traced with cholera toxin B in sagittal sections following SCI (dorsal hemisection) or conditioning lesion (CL) in rats; r: rostral; c: caudal; d: dorsal; v: ventral. Dashed lines label the border of the glial scar. Scale bar: 100 μm. **(B)** Representative images of neurite outgrowth of naïve and conditioned rat DRG neurons (CL). Scale bar: 50 μm. **(C)** Quantification of B; (n = 40 to 92). **(D)** Western blot analysis of P-AktSer473, P-GSK3βSer9 and P-GSK3βTyr216 on DRG extracts from uninjured rats (Un) or subjected to SCI or CL. **(E)** Quantification of D; (n = 3 to 4). **(F)** Western blot analysis of P-GSK3βSer9 and P-GSK3βTyr216 in SCI site extracts from rats with SCI or conditioning lesion (CL); **(G)** Quantification of F; (n = 5 to 7). **(H)** P-GSK3βTyr216 and GSK3β immunostaining of the growth cone of naïve rat DRG neurons or conditioned (CL) neurons; (n = 10 to 12). Scale bar: 4 μm. **(I)** Western blot analysis of P-CRMP-2 on SCI site extracts from rats with SCI or conditioning lesion (CL); (n = 4 to 7). **(J)** Western blot analysis of P-FynTyr530 on SCI site extracts from rats with SCI or conditioning lesion (CL); (n = 4 to 5). **(K)** P-GSK3βTyr216 and GSK3β staining of the growth cone of control transfected DRG neurons or neurons transfected with a Fyn kinase dead construct (Fyn KD); (n = 72 to 101). Scale bar: 3 μm. All error bars are SEM. *P <0.05. **P <0.01. ***P <0.001. Two-tailed Student’s t test. CRMP-2, collapsin response mediator protein 2; DRG, dorsal root ganglia; GSK3β, glycogen synthase kinase 3β; SCI, spinal cord injury; SEM, standard error of the mean.

We used the conditioning lesion model [1,18] to identify mechanisms promoting axonal regeneration. For that we analyzed DRGs from rats with either SCI or conditioning lesion using a phospho-site broad signaling pathway screen (Kinexus) that examines 38 phosphorylation sites in 32 key signaling proteins. In the CL group, SCI was preceded one week by a sciatic nerve transection. As previously described [1], rats with conditioning lesion had an increased number of dorsal column axons entering the glial scar (Figure 1A), with some axons being capable of regenerating beyond the rostral border of the scar. As expected [1,18], the conditioning effect was also observed *in vitro* as conditioned DRG neurons (CL; neurons isolated from rats with a sciatic nerve transection performed one week prior to DRG removal) had an increased neurite outgrowth (Figure 1B,C). The analysis of the phospho-site broad signaling pathway screen revealed that following conditioning lesion increased P-AktSer473 (activated Akt), increased P-GSK3βSer9 (which negatively regulates GSK3β activity), and decreased P-GSK3βTyr216 were present (Table 1). No differential phosphorylation of GSK3α was found (Table 1). Combined, these observations indicate that following a conditioning lesion there is an overall decrease of GSK3β activity. Validation was performed by Western blot in DRG (Figure 1D,E,) where we also analyzed samples from uninjured animals. After conditioning lesion, increased P-AktSer473 was found relative to both uninjured DRG and DRG collected after SCI (Figure 1D,E). Regarding P-GSK3βSer9, although after SCI the levels were increased relative to uninjured DRG, a more pronounced increase was observed after conditioning lesion (Figure 1D,E). The levels of P-GSK3βTyr216 were decreased after conditioning lesion, when compared to both uninjured DRG and DRG collected after SCI (Figure 1D,E). Locally at the SCI site no differences were found in P-GSK3βSer9, while P-GSK3βTyr216 was decreased after conditioning lesion (Figure 1F,G). Accordingly, in the growth cone of conditioned DRG neurons a 1.9-fold decreased ratio (*P* <0.0001) of P-GSK3βTyr216/GSK3βwas present (Figure 1H). The decreased P-GSK3βTyr216 levels at the SCI site of rats with conditioning lesion correlated with a 1.5-fold decrease in GSK3β kinase activity (*P* <0.05) and decreased phosphorylation of CRMP-2 (Figure 1I).

**Table 1:** Proteins from the GSK3β pathway differentially regulated in DRG after CL when compared to SCI

	**CL/SCI**
Protein kinase B alpha (Akt) [S473]	1,53
Glycogen synthase kinase 3 beta (GSK3β) [S9]	1,55
Glycogen synthase kinase 3 beta (GSK3β) [Y216]	0,53
Glycogen synthase kinase 3 alpha (GSK3α) [S21]	0,95
Glycogen synthase kinase 3 alpha (GSK3α) [Y279]	1,04

SCI, spinal cord injury; CL, conditioning lesion; DRG, dorsal root ganglia.

Initial GSK3βTyr216 phosphorylation occurs through an autocatalytic event [8], although the upstream kinases Pyk2 and Fyn can also target this residue [9,10]. Whereas we did not detect Pyk2 either in the DRG or spinal cord, the inactive form of Fyn kinase, P-FynTyr530, was increased after conditioning lesion (Figure 1J; *P* <0.01). Moreover, DRG neurons transfected with Fyn kinase dead (Fyn Lys299Met) had a 1.6-fold decreased ratio (*P* <0.0001) of P-GSK3βTyr216/GSK3β (Figure 1K) in support of a Fyn kinase-mediated phosphorylation of GSK3βTyr216 under these conditions. Besides phosphorylation by Fyn kinase, a 1.3-fold higher phosphatase activity against P-GSK3βTyr216 was found in animals with conditioning lesion (*P* <0.05) further supporting a tight control of the P-GSK3βTyr216 levels, probably achieved by dual regulation of upstream kinases and phosphatases.

### Enhanced axonal growth in conditioned DRG neurons is related to increased microtubule growth speed in the growth cone

**Figure 2:**
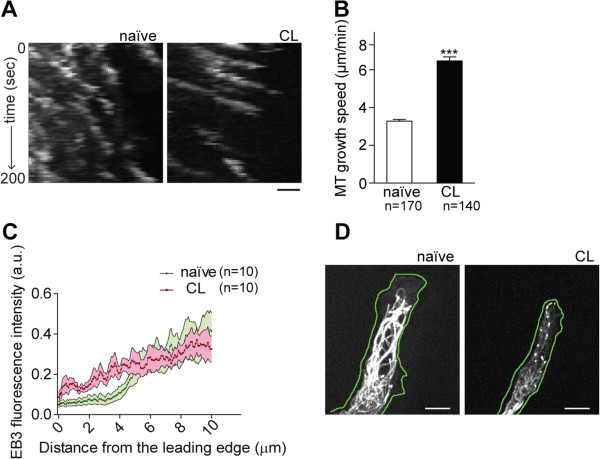
**Microtubule growth speed is increased in the growth cone of conditioned DRG neurons. (A)** Kymographs of single processes of naïve and conditioned (CL) neurons transfected with EB3-GFP. Scale bar: 1 μm. **(B)** Quantification of microtubule growth speed in naïve and in conditioned (CL) neurons transfected with EB3-GFP; (n = 140 to 170 microtubules). **(C)** Quantification of EB3-fluorescence intensity relative to the distance to the growth cone tip in naïve and in conditioned (CL) neurons transfected with EB3-GFP; (n = 10 growth cones). For distances closer to the leading edge (between 0.48 and 4.48 μm) the differences presented are statistically significant with *P* <0.05. **(D)** Representative images of growth cones of naïve and in conditioned (CL) neurons transfected with EB3-GFP. The green line surrounds the leading edge of the cell. Scale bar: 5 μm. All error bars are SEM. ***P <0.001. Two-tailed Student’s t test. CL, conditioned lesion; DRG, dorsal root ganglia; SEM, standard error of the mean.

Our results showed a downregulation of GSK3β activity through decreased GSK3βTyr216 phosphorylation in conditioned neurons. Given the involvement of GSK3β in microtubule dynamics, the microtubule growth rate in naïve and conditioned rat DRG neurons was measured. For that, we visualized polymerizing microtubule ends by transfecting neurons with the plus-tip binding protein EB3. When compared to naïve neurons, after conditioning lesion, EB3 comets moved with a 1.8-fold increased speed (Figure 2A,B, see also Additional file 1: Movie S1). Increased microtubule growth speed in conditioned neurons [see Additional file 1: Movie S1] correlated with increased neurite outgrowth (Figure 1C). Moreover, by extending the imaging periods, increased axon elongation and increased microtubule growth speed were observed in conditioned neurons, when compared to naïve neurons [see Additional file 2: Movie S2 and Additional file 3: Figure S1]. Regarding the number of growing microtubules, no differences were observed between naïve and conditioned DRG neurons (data not shown). Moreover, in accordance with an increased microtubule growth speed in the growth cone, we observed that the EB3 fluorescence was located within a shorter distance from the growth cone tip in conditioned neurons (Figure 2C,D). This suggests that in conditioned neurons, growth of microtubules into the peripheral domain of the growth cone is promoted. Overall, conditioned DRG neurons exhibited decreased GSK3β activity through downregulation of P-GSK3βTyr216 and increased microtubule growth speed and growth into the peripheral domain of the growth cone.

### *In vivo*, inhibition of GSK3β via Ser9 phosphorylation is dispensable for gain of axonal regeneration capacity

**Figure 3:**
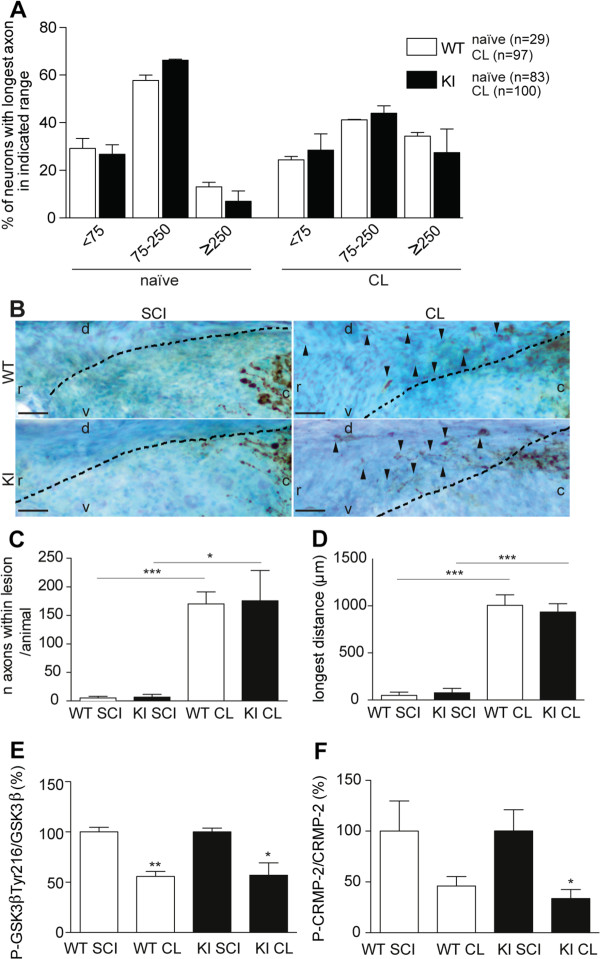
***In vivo*, inhibition of GSK3β via Ser9 phosphorylation is dispensable for gain of axonal regeneration capacity. (A)** Quantification of neurite outgrowth, measured as the percentage of neurons with the longest axon within each range (<75 μm, 75 to 250 μm, or ≥250 μm), of naïve and conditioned (CL) DRG neurons from either WT or GSK3βSer9Ala KI mice (KI); (n = 29 to 100). **(B)** Representative images of CT-B + fibers in sagittal spinal cord sections following SCI (left panels) or conditioning lesion (right panels) in WT (upper panels) and GSK3βSer9Ala KI mice (lower panels). r: rostral; c: caudal; d: dorsal; v: ventral; arrowheads highlight axons regenerating rostrally to the SCI site. Dashed lines label the border of the glial scar. Scale bar: 50 μm. **(C)** Quantification of the number of CT-B + dorsal column fibers able to enter the glial scar; (n = 4 to 6). **(D)** Quantification of the longest distance of regeneration of CT-B + dorsal column fibers scored from the border of the lesion; (n = 4 to 6). **(E)** Quantification of P-GSK3βTyr216 in relation to total GSK3β, as assessed by western blot, in either WT or GSK3βSer9Ala KI mice subjected to SCI (WT SCI or KI SCI) or conditioning lesion (WT CL or KI CL). Results are presented as a percentage of the SCI condition. **(F)** Quantification of P-CRMP-2 in relation to total CRMP-2, as assessed by western blot, in either WT or GSK3βSer9Ala KI mice subjected to SCI (WT SCI or KI SCI) or conditioning lesion (WT CL or KI CL); (n = 3 to 4). Results are presented as a percentage of the SCI condition. All error bars are SEM. *P <0.05. **P <0.01. ***P <0.001. Two-tailed Student’s t test. CRMP-2, collapsin response mediator protein 2; DRG, dorsal root ganglia; GSK3β, glycogen synthase kinase 3β; SCI, spinal cord injury; SEM, standard error of the mean; WT, wild type.

Given that Ser9 has been widely considered the key regulatory residue of GSK3β [12], we used GSK3βSer9Ala knockin (KI) mice [16] to further assess its role *in vivo* during axonal regeneration. In these animals, the endogenous Ser9 codon of GSK3β was changed to encode the non-phosphorylatable Ala residue [16]. GSK3β activity in the spinal cord of GSK3βSer9Ala KI mice was increased 1.8-fold when compared to wild type (WT) mice (*P* <0.05), in agreement with the known inhibitory activity of Ser9 phosphorylation. *In vitro*, naïve GSK3βSer9Ala KI DRG neurons displayed a similar neurite outgrowth to that of naïve WT neurons and the percentage of longer neurites was increased following conditioning lesion (Figure 3A). *In vivo*, tracing of dorsal column fibers with cholera toxin subunit B (CT-B) after a spinal cord dorsal hemisection corroborated the *in vitro* data, with GSK3βSer9Ala KI axons displaying a similar behavior to that of WT axons (Figure 3B-D). For both genotypes, we observed negligible axonal growth through the glial scar following SCI, whereas an increased number of axons entering the glial scar able to regenerate for longer distances were seen following conditioning lesion (Figure 3B-D). Neither WT nor GSK3βSer9Ala KI mice showed axons extending rostrally beyond the glial scar, as the conditioning effect is less robust in mice than in rats [19]. These findings demonstrate that modulation of GSK3β through Ser9 phosphorylation is not required to induce axonal growth. Moreover, in the SCI site, both WT and GSK3βSer9Ala KI mice were able to regulate GSK3β activity through reduced phosphorylation of Tyr216 after conditioning lesion (Figure 3E). In accordance with decreased GSK3β kinase activity after conditioning lesion, the levels of P-CRMP-2 were decreased in the SCI site of both WT and GSK3βSer9Ala KI mice (Figure 3F). These results further support that downregulation of GSK3β activity through decreased Tyr216 phosphorylation represents the primary regulatory event leading to increased axonal growth.

### Partial reduction of GSK3β activity increases axonal growth through the glial scar

**Figure 4:**
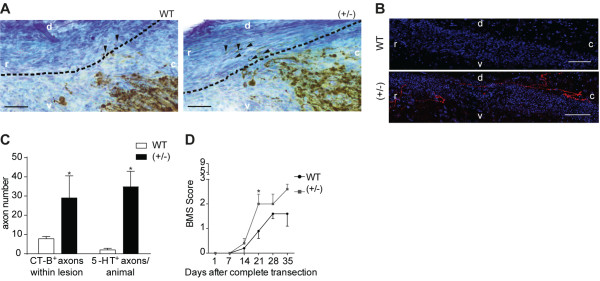
**Partial reduction of GSK3β activity increases axonal growth through the glial scar. (A)** Representative images of dorsal column fibers traced with CT-B in sagittal sections following dorsal hemisection in WT or GSK3β(+/-) mice. r: rostral; c: caudal; d: dorsal; v: ventral. Dashed lines label the border of the glial scar. Arrowheads highlight axons regenerating rostrally to the SCI site. Scale bar: 50 μm. **(B)** Representative images of 5-HT-immunostained serotonergic axons (red) counterstained with DAPI (blue) observed caudal to the lesion site in sagittal spinal cord sections following a complete transection in WT or GSK3β(+/-) mice; (n = 5 to 6). Scale bar: 50 μm. **(C)** Number of CT-B + dorsal column axons able to enter the glial scar following dorsal hemisection (CT-B+) and of 5-HT-immunostained serotonergic axons observed caudal to the lesion site following a complete transection (5-HT+) in WT or GSK3β(+/-) mice. **(D)** Basso Mouse Scale (BMS) open field test following a complete spinal cord transection in WT and GSK3β(+/-) mice; (n = 6 to 7). All error bars are SEM. *P <0.05. Two-tailed Student’s t test. DAPI, 4′,6-diamidino-2-phenylindole; GSK3β, glycogen synthase kinase 3β; SCI, spinal cord injury; SEM, standard error of the mean; WT, wild type; 5-HT, 5-hydroxytryptamine.

To further demonstrate that decreased GSK3β activity is related to increased axonal growth, we assessed axonal regeneration in GSK3β knockout heterozygous mice (GSK3β (+/-)) [17]. As expected, GSK3β (+/-) mice exhibit an approximately 50% reduction of GSK3β kinase activity in the spinal cord (*P* <0.05). Assessment of axonal regeneration of ascending dorsal column fibers after a dorsal spinal cord hemisection by tracing with CT-B (Figure 4A) showed that GSK3β (+/-) mice have an increased number of axons inside the glial scar (Figure 4C) and that these are capable of regenerating for longer distances (WT: 39.0 ± 7.8, and GSK3β (+/-): 111.7 ± 32.4 μm; *P* <0.05). The conditioning lesion is a very useful model to understand mechanisms underlying central axon regeneration and several molecules initially identified using this paradigm were later found to increase regeneration of other unrelated tracts. To evaluate whether downregulation of GSK3β activity might promote axonal regeneration of other tracts, regeneration of serotonergic axons was assessed. 5-Hydroxytryptamine (5-HT) immunostaining of descending raphespinal serotonergic axons after complete spinal cord transection (Figure 4B) showed that the number of 5-HT-positive axons caudal to the lesion site was increased 17.4-fold in GSK3β(+/-) mice (Figure 4C). This demonstrates that in a non-sensory tract, lower levels of GSK3β increase axonal regeneration through and beyond the glial scar. Of note, the basal levels of 5-HT immunostaining in uninjured spinal cords were similar between WT and GSK3β(+/-) mice (data not shown). The increased regeneration of GSK3β(+/-) serotonergic axons correlated with an improved recovery of open field locomotor activity (Figure 4D), as expected given the modulation by serotonergic axons of the activity of spinal motor systems [20]. Uninjured mice of both genotypes displayed similar maximum locomotor scores. We excluded the possibility that differences in axonal regeneration in GSK3β(+/-) mice might be related to stimulation of astrocyte migration [21] as the glial scar, assessed by the glial fibrillary acidic protein (GFAP)-negative area, remained similar between the two strains (data not shown). These results show that partial reduction of GSK3β increases axonal growth following SCI.

### Neuronal deletion of GSK3β promotes axonal regeneration in the CNS

**Figure 5:**
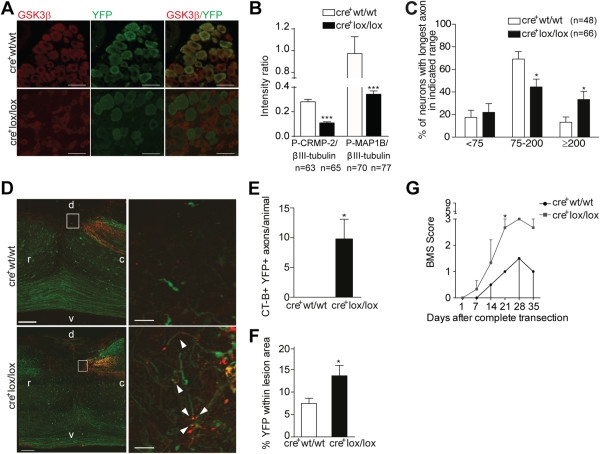
**Neuronal GSK3β deletion increases axonal growth. (A)** Representative images of GSK3β immunohistochemistry in DRG neurons from cre^+^GSK3βwt/wt (cre^+^wt/wt) mice and cre^+^GSK3βlox/lox (cre^+^lox/lox). Scale bar: 50 μm. **(B)** Quantification of the intensity ratio of the P-CRMP-2/βIII-tubulin and P-MAP1B/βIII-tubulin immunostainings depicted in C; CRMP-2/βIII-tubulin (n = 63 to 65 growth cones) and P-MAP1B/βIII-tubulin (n = 70 to 77 growth cones). **(C)** Neurite outgrowth in naïve DRG neurons from cre^+^GSK3βwt/wt and cre^+^GSK3βlox/lox mice quantified as the percentage of neurons with the longest axon within each range: <75 μm, 75 to 200 μm, or ≥200 μm; (n = 48 to 66). **(D)** Representative images of sagittal spinal cord sections following dorsal hemisection, from cre^+^GSK3βwt/wt and cre^+^GSK3βlox/lox mice. YFP^+^ axons are shown in green and dorsal column fibers traced with CT-B are labeled in red; higher magnifications of cre^+^GSK3βwt/wt and cre^+^GSK3βlox/lox showing the YFP^+^/CT-B^+^ axons (arrowheads) within the glial scar are shown on the right. r: rostral; c: caudal; d: dorsal; v: ventral. Scale bar: 25 μm. **(E)** Number of CT-B^+^YFP^+^ axons able to enter the glial scar in cre^+^GSK3βwt/wt and cre + GSK3βlox/lox mice; (n = 4 to 5). **(F)** Percentage of lesion area occupied by YFP^+^ axons (% YFP within lesion area) in cre^+^GSK3βwt/wt and cre^+^GSK3βlox/lox mice; (n = 4 to 5). **(G)** Locomotor recovery in cre^+^GSK3βwt/wt and cre^+^GSK3βlox/lox mice assessed by Basso Mouse Scale following complete spinal cord transection. All error bars are SEM. *P <0.05. ***P <0.001. Two-tailed Student’s t test. CRMP, collapsin response mediator protein; GSK3β, glycogen synthase kinase 3β; MAP1B, microtubule-associated protein 1B; SEM, standard error of the mean; YFP, yellow fluorescent protein.

To avoid possible confounding effects of GSK3β depleted glia, we used mice with an inducible GSK3β neuronal deletion (cre^+^GSK3βlox/lox mice) generated by crossing GSK3βlox/lox [22] with Slick-H mice (that is, mice that co-express both inducible-CreER^T2^ and yellow fluorescent protein (YFP) under the control of the Thy1 promoter) [23]. In these animals cre-positive neurons are YFP-positive. In the cre^+^GSK3βlox/lox mice, GSK3β was deleted in 97% of the YFP^+^ DRG neurons (Figure 5A), which represented 40% of all DRG neurons. We did not observe any compensation by alterations in expression of the GSK3α isoform in the YFP^+^ cells of cre^+^GSK3βlox/lox mice (data not shown). As expected, growth cones of GSK3β depleted neurons had residual levels of P-CRMP-2 and P-MAP1B (Figure 5B), probably accounted for by phosphorylation by alternative kinases.

*In vitro*, DRG neurons from cre^+^GSK3βlox/lox mice had increased neurite outgrowth with more neurons extending longer neurites (≥200 μm) (Figure 5C). *In vivo*, after dorsal spinal cord hemisection, tracing with CT-B of YFP^+^ dorsal column fibers corroborated the *in vitro* findings, as cre^+^GSK3βlox/lox mice had a 9.8-fold increased number of CT-B+/YFP^+^ axons entering the glial scar (Figure 5D, E). Moreover, the area of the lesion occupied by YFP^+^ axons was increased 1.8-fold in cre^+^GSK3βlox/lox (Figure 5D,F). Since following dorsal hemisection all the dorsal column fibers were transected, CT-B^+^/YFP^+^ axons entering the glial scar reflect axonal regeneration. In the case of CT-B^-^/YFP^+^ axons, we cannot discard increased collateral sprouting arising from uninjured axons. We did not assess regeneration of raphespinal serotonergic axons as Slick-H mice do not express Cre recombinase in this tract. Although unrelated to an *in vivo* regenerative effect of DRG axons, after complete spinal cord transection, open field locomotor recovery was increased in cre^+^GSK3βlox/lox mice (Figure 5G), further supporting that neuronal deletion of GSK3β promotes regeneration and/or sprouting of other tracts besides the dorsal column tract. Uninjured mice of both genotypes displayed similar maximum locomotor scores. These data indicate that total deletion of GSK3β in neurons increases axonal regeneration in the CNS within the glial scar.

### Decreased GSK3β activity leads to increased microtubule growth speed in the growth cone

**Figure 6:**
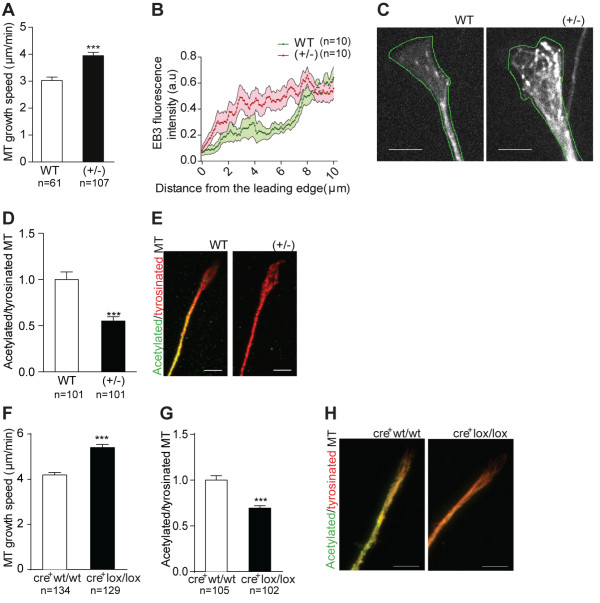
**Decreased GSK3β activity leads to increased microtubule dynamics in the growth cone. (A)** Quantification of microtubule growth speed in naïve DRG neurons from WT or GSK3β(+/-) mice transfected with EB3-GFP; (n = 61 to 107 microtubules). **(B)** Quantification of EB3-fluorescence intensity relative to the distance to the tip of the growth cone, in naïve DRG neurons from WT or GSK3β(+/-) mice transfected with EB3-GFP; (n = 10 growth cones). For distances between 0.80 to 1.12, 2.64 to 3.04, 4.64 to 7.04 μm the differences shown are statistically significant with *P* <0.05. **(C)** Representative images of growth cones of naïve DRG neurons from WT or GSK3β(+/-) mice transfected with EB3-GFP. The green line surrounds the leading edge of the cell. Scale bar: 4 μm. **(D)** Quantification of the intensity ratios between acetylated and tyrosinated microtubules in WT or GSK3β(+/-) growth cones; (n = 101 growth cones). **(E)** Representative images of growth cones of naïve DRG neurons from WT or GSK3β(+/-) mice immunostained with acetylated and tyrosinated α-tubulin. Scale bar: 5 μm. **(F)** Quantification of microtubule growth speed in naïve neurons from cre^+^GSK3βwt/wt mice or cre^+^GSK3βlox/lox mice transfected with EB3-mCherry (n = 117 to 134 microtubules). **(G)** Quantification of the intensity ratios between acetylated and tyrosinated microtubules in cre^+^GSK3βwt/wt or cre^+^GSK3βlox/lox growth cones; (n = 102 to 105 growth cones). **(H)** Representative images of growth cones from cre^+^GSK3βwt/wt or cre^+^GSK3βlox/lox mice immunostained for acetylated and tyrosinated α-tubulin. Scale bar: 5 μm. All error bars are SEM. ***P <0.001. Two-tailed Student’s t test. DRG, dorsal root ganglia; GFP, green fluorescent protein; GSK3β, glycogen synthase kinase 3β; SEM, standard error of the mean. WT, wild type.

To establish a causal effect between decreased GSK3β activity and altered microtubule dynamics in the growth cone, we measured microtubule growth speed in GSK3β(+/-) DRG neurons. GSK3β(+/-) neurons had an increased microtubule growth speed in the growth cone (Figure 6A, see also Additional file 4: Movie S3) which correlated with an increased axonal growth as seen by extending the periods of imaging [see Additional file 5: Movie S4 and Additional file 6: Figure S2], similar to what was observed in conditioned DRG neurons (Figure 2B). With respect to the number of EB3 comets, no differences were observed (data not shown). Moreover, GSK3β(+/-) neurons presented EB3 fluorescence located within a shorter distance from the growth cone tip (Figure 6B,C), supporting an increased growth of microtubules into the peripheral domain of the growth cone. Accordingly, a decreased ratio of acetylated (stable)/tyrosinated (dynamic) microtubules in the growth cones of GSK3β(+/-) neurons was found (Figure 6D,E). In DRG neurons from cre^+^GSK3βlox/lox mice, increased microtubule growth speed was present in the growth cone (Figure 6F, see also Additional file 7: Movie S5) and also correlated with increased axonal growth (Figure 5C, see also Additional file 8: Movie S6 and Additional file 9: Figure S3). Moreover, cre^+^GSK3βlox/lox neurons also presented a decreased ratio of acetylated/tyrosinated microtubules (Figure 6G,H). Of note, whereas with GSK3β(+/-) neurons EB3-GFP was used, with cre^+^ GSK3βlox/lox neurons EB3-mCherry was employed as cre^+^ neurons are YFP^+^. The differences in these constructs (details available in the Methods), might underlie the differences found among controls (WT and cre^+^GSK3βwt/wt) for the parameters assessed. In summary, these results demonstrate that either partial reduction or total deletion of GSK3β increases microtubule dynamics in the growth cone that correlates with increased axon growth.

### Increased microtubule growth speed in the growth cone of GSK3β depleted neurons is related to decreased phosphorylation of CRMP-2

**Figure 7:**
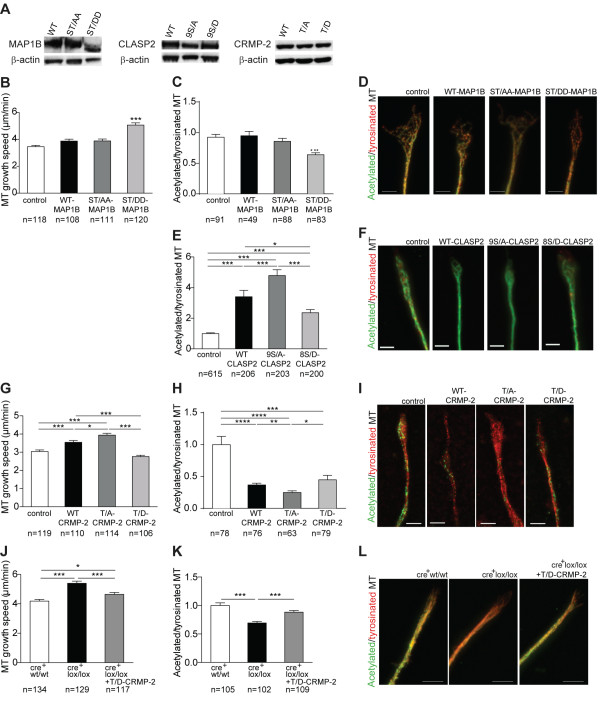
**Increased microtubule growth in GSK3β depleted neurons is related to decreased CRMP-2 phosphorylation. (A)** MAP1B, CLASP2 and CRMP-2 western in CAD cells. **(B)** Microtubule growth in rat neurons transfected with EB3-mCherry alone (control) or co-transfected with WT MAP1B, ST/AA-MAP1B or ST/DD-MAP1B. **(C)** Intensity ratios between acetylated and tyrosinated microtubules in growth cones from rat DRG neurons transfected with EB3-mCherry alone (control) or co-transfected with WT MAP1B, ST/AA-MAP1B or ST/DD-MAP1B. **(D)** Representative images of C. Scale bar: 3 μm. **(E)** Intensity ratios between acetylated and tyrosinated microtubules in growth cones from rat naïve DRG neurons either non-infected (control) or infected with WT CLASP2, 9S/A-CLASP2 or 8S/D-CLASP2. **(F)** Representative images of E. Scale bar: 3 μm. **(G)** Microtubule growth in neurons transfected with EB3-GFP alone (control) or co-transfected with WT CRMP-2, T/A-CRMP-2, or T/D-CRMP-2. **(H)** Intensity ratios between acetylated and tyrosinated microtubules in growth cones from neurons transfected with EB3-GFP alone (control) or co-transfected with WT CRMP-2, T/A-CRMP-2, or T/D-CRMP-2. **(I)** Representative images of H. Scale bar: 5 μm. **(J)** Microtubule growth in naïve neurons from cre^+^GSK3βwt/wt mice or cre+GSK3βlox/lox mice transfected with EB3-mCherry, or cre^+^GSK3βlox/lox mice co-transfected with EB3-mCherry and T/D-CRMP-2 (cre^+^GSK3βlox/lox + T/D-CRMP-2). **(K)** Intensity ratios between acetylated and tyrosinated microtubules in growth cones from cre^+^GSK3βwt/wt, cre+GSK3βlox/lox or cre^+^GSK3βlox/lox transfected with T/D-CRMP-2 (cre^+^GSK3βlox/lox + T/D-CRMP-2). **(L)** Representative images of K. Scale bar: 5 μm. All error bars are SEM. *P <0.05. **P <0.01. ***P <0.001. ****P <0.0001. Two-tailed Student’s t test. CLASP2, cytoplasmic linker associated protein 2; CRMP, collapsin response mediator protein; DRG, dorsal root ganglia; GSK3β, glycogen synthase kinase 3β; MAP1B, microtubule-associated protein 1B; SEM, standard error of the mean. WT, wild type.

To determine the mechanism underlying the effect of GSK3β in microtubule growth speed, we analyzed the role of key GSK3β substrates, namely MAP1B, CLASP2 and CRMP-2. In DRG neuron cultures, the high percentage of satellite cells and the low efficiency of nucleofection and transduction, preclude a straightforward comparison between endogenous and overexpressed protein levels. To allow the comparison of the relative expression of the different mutants analyzed, transfection (for MAP1B and CRMP-2) or transduction (for CLASP2) of a neuronal cell line was performed and revealed similar levels of expression among different mutants (Figure 7A).

Phosphorylation of MAP1B by GSK3β increases microtubule dynamics in non-neuronal cells [24,25]. In neurons, overexpression of either WT MAP1B or of the phospho-resistant mutant ST/AA-MAP1B had no significant impact in microtubule growth speed or in the ratio of acetylated/tyrosinated microtubules (Figure 7B-D, see also Additional file 10: Movie S7). However, the phospho-mimetic mutant ST/DD-MAP1B increased microtubule growth speed and led to a decreased ratio of acetylated/tyrosinated microtubules (Figure 7B-D, see also Additional file 10: Movie S7). As such, P-MAP1B in neurons is sufficient but not necessary to increase microtubule dynamics, as in GSK3β depleted neurons, reduced levels of P-MAP1B (Figure 5C) and increased microtubule growth speed co-exist.

CLASP2 is a microtubule plus end-binding protein that when overexpressed in COS cells, promotes microtubule stabilization [26]. GSK3β phosphorylates CLASP2 at multiple sites in the domain required for microtubule plus end tracking, disrupting CLASP2 association with microtubules [27]. DRG neurons overexpressing WT CLASP2 had an increased ratio of acetylated/tyrosinated microtubules (Figure 7E,F). Viral transduction with CLASP2 followed by transfection with EB3 rendered neurons unhealthy for subsequent analyses of microtubule growth speeds. The phospho-resistant mutant 9S/A-CLASP2 promoted an increase in the ratio of acetylated/tyrosinated microtubules more pronounced than WT CLASP2, whereas the phospho-mimetic mutant 8S/D-CLASP2 showed the weakest effect (Figure 7E,F). These results agree with the function of CLASP2 in promoting microtubule stability, which is inhibited by GSK3β phosphorylation. As such, the altered microtubule stability in GSK3β depleted neurons is unrelated to decreased P-CLASP2, suggesting that an alternative GSK3β substrate is responsible for this effect.

CRMP-2 binds to tubulin heterodimers to promote microtubule assembly, thereby enhancing axon elongation, and its microtubule-binding activity is decreased upon GSK3β phosphorylation [28]. In neurons, overexpression of WT CRMP-2 or of the phospho-resistant mutant T/A-CRMP-2 led to increased microtubule growth speed (Figure 7G, see also Additional file 11: Movie S8), which was reversed by transfection with the phospho-mimetic mutant T/D-CRMP-2 (Figure 7G, see also Additional file 11: Movie S8). Moreover, overexpression of CRMP-2 decreased the ratio of acetylated/tyrosinated microtubules; this effect being stronger when a phospho-resistant mutant (T/A-CRMP-2) was used and less pronounced with the phospho-mimetic mutant T/D-CRMP-2 (Figure 7H,I). These results demonstrate that in neurons, the binding of CRMP-2 to tubulin heterodimers in the growth cone controls the rate of microtubule assembly and that this effect is negatively regulated by GSK3β phosphorylation. Moreover, overexpression of T/D-CRMP-2 in cre^+^GSK3βlox/lox neurons reversed their increased microtubule growth speed (Figure 7J, see also Additional file 7: Movie S5), which correlated with a decreased axon growth [see Additional file 8: Movie S6 and Additional file 9: Figure S3] and a decreased ratio of acetylated/tyrosinated microtubules (Figure 7K,L). Combined, these results demonstrate that neuronal deletion of GSK3β alters the microtubule cytoskeleton in the growth cone, specifically by regulating the levels of P-CRMP-2.

### Inactivation of the GSK3β-CRMP-2 pathway participates in the conditioning effect attenuating the growth repression mediated by CNS inhibitors

**Figure 8:**
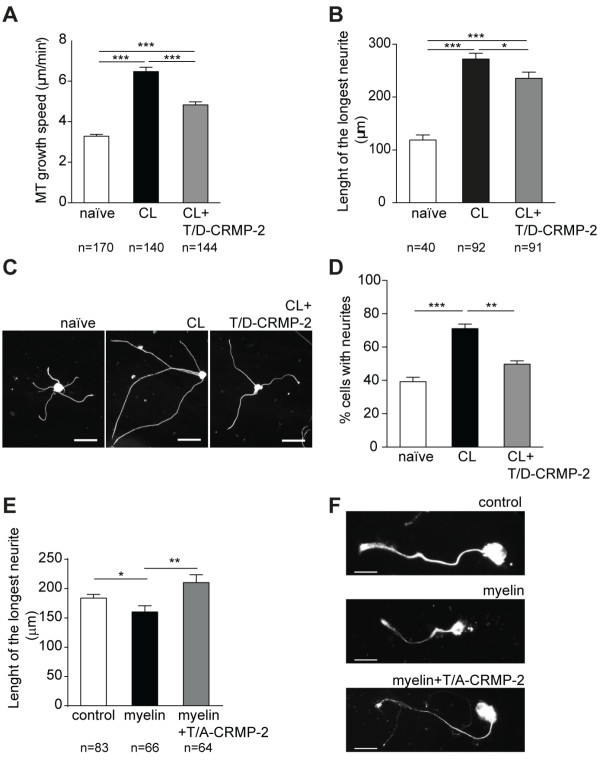
**The GSK3β-CRMP-2 pathway participates in the conditioning lesion effect. (A)** Quantification of microtubule growth speed in naïve and conditioned (CL) DRG neurons transfected with EB3-GFP, or conditioned neurons co-transfected with EB3-GFP and T/D-CRMP-2 (CL + T/D-CRMP-2); (n = 140 to 170 microtubules). **(B)** Quantification of the average size of the longest neurite in naïve and conditioned (CL) DRG, or conditioned neurons overexpressing T/D-CRMP-2 (CL + T/D-CRMP-2); (n = 40 to 91). **(C)** Representative images of C. Scale bar: 100 μm. **(D)** Quantification of the percentage of cells with neurites in naïve and conditioned (CL) DRG, or conditioned neurons overexpressing T/D-CRMP-2 (CL + T/D-CRMP-2). **(E)** Quantification of the size of the longest neurite of DRG neurons plated on top of laminin (control) or laminin + myelin (myelin), either transfected with pEGFP-C1 or co-transfected with pEGFP-C1 and T/A-CRMP-2 (myelin + T/A-CRMP-2); (n = 64 to 83). **(F)** Representative images of F. Scale bar: 30 μm. All error bars are SEM. *P <0.05. **P <0.01. ***P <0.001. One way ANOVA. ANOVA, analysis of variance; CRMP, collapsin response mediator protein; DRG, dorsal root ganglia; GFP, green fluorescent protein; GSK3β, glycogen synthase kinase 3β; SEM, standard error of the mean.

In conditioned DRG neurons, enhanced axonal growth and increased microtubule growth speed in the growth cone are present. At the molecular level, we determined that inhibition of GSK3β activity and decreased levels of P-CRMP-2 occurred in this model. To further demonstrate that the GSK3β-CRMP-2 pathway is critical for the conditioning effect, we transfected conditioned DRG neurons with the phospho-mimetic mutant T/D-CRMP-2. Overexpression of phospho-mimetic CRMP-2 partially reversed the increased microtubule growth speed of conditioned neurons (Figure 8A, see also Additional file 1: Movie S1) and this effect correlated with a decrease in neurite outgrowth (Figure 8B,C, see also Additional file 2: Movie S2 and Additional file 3: Figure S1). A pronounced reversion of the conditioning effect was observed at the level of neurite formation as overexpression of T/D-CRMP-2 in conditioned neurons led to a decrease in the percentage of cells with neurites to levels similar to those observed in naïve neurons (Figure 8D). We next assessed whether reducing the levels of P-CRMP-2 by overexpression of T/A-CRMP-2 affected myelin-mediated inhibition of neurite outgrowth in naïve DRG neurons. As expected, purified CNS myelin decreased the growth of the longest DRG neurite (Figure 8E,F). Interestingly, overexpression of phospho-resistant T/A-CRMP-2 counteracted the inhibitory effect of myelin (Figure 8E,F). Together these results demonstrate that inactivation of the GSK3β-CRMP-2 pathway participates in the conditioning effect overcoming the growth repression mediated by CNS inhibitors.

## Discussion

A conditioning lesion reprograms neurons to a regenerative status. So far, none of the molecules identified as playing a role in this paradigm was shown to be sufficient and necessary to mimic the gain of axonal regenerative capacity elicited, probably as it is obtained through the combination of multiple components. As such, the quest for clinically relevant molecules that contribute to the conditioning lesion effect should be pursued. In this work, we show that the GSK3β-CRMP-2 pathway participates in this paradigm, with decreased GSK3β activity and phospho-CRMP-2 levels promoting axonal regeneration and attenuating the growth repression mediated by CNS inhibitors. The effect of a conditioning lesion in increasing the microtubule growth speed is more robust than that of GSK3β deletion alone, which supports that other pathways might participate in this effect, as is the case in HDAC5 activation [5].

At the mechanistic level, we demonstrate that *in vivo*, both in WT animals and by blocking Ser9 phosphorylation, GSK3β inhibition occurs through regulation of P-Tyr216 levels. A dual mechanism for the regulation of phosphorylated GSK3βTyr216 is probably in place after SCI, as increased specific phosphatase activity and decreased Fyn kinase levels are observed in conditions of increased axonal growth. In contrast to our data, after nerve crush injury, both GSK3β P-Ser9 and P-Tyr216 have been reported to be increased both in ipsilateral and contralateral nerves, which causes difficulties in the interpretation of the results [29]. Recently, regulation of axon regeneration through PI3K-GSK3 signaling, occurring through induction of Smad1 expression, was shown to be specific in the soma but dispensable for local axon assembly in the distal axons. Interestingly, this suggested the existence of upstream regulators responsible for modulating GSK3 signaling in growth cones of adult sensory neurons [30]. In this respect, we now show that regulation of P-GSK3βTyr216 levels, achieved by the dual action of upstream kinases and phosphatases, is a possible mechanism responsible for modulating GSK3 activity specifically in growth cones.

Although several studies attempted to understand the role of GSK3β in axon growth and regeneration, as recently reviewed [31], most of the reports used cultured neurons or neuronal cell lines, and semi-specific pharmacological inhibitors, yielding conflicting results ranging from promotion of axon growth to axon growth inhibition. Some *in vitro* studies suggested that reduced GSK3β activity is required for axon formation, elongation or decreased retraction [28,32-36], in a CRMP-2-dependent mechanism [28,36]. Accordingly, *in vivo* studies using GSK3β inhibitors showed increased axon regeneration following spinal cord injury [14]. Supporting that *in vivo* GSK3β inactivation promotes axon regeneration, acutely blocking PI3K signaling or depleting Smad1 in adult mice prevented sensory axon regeneration [30]. Along the same line, other studies suggested that increased GSK3β activity leads to neurite retraction [37]. However, a second group of studies reported opposite effects supporting that increased GSK3β activity leads to increased axon elongation. In this respect, high phosphorylation of GSK3 targets (including CMRP-2) was shown to increase neurite elongation [38-40] and pharmacological inhibition or small hairpin RNA (shRNA) blockage of GSK3β activity was reported to induce neurite retraction [15,41,42], with participation of CRMP-4 in the inhibitory effects [15]. The discrepancies in the literature were reconciled with the concept that the final outcome of GSK3β inhibition would depend on the extent of inhibition. In this paradigm, partial inactivation of GSK3β in the growth cone would lead to efficient axonal elongation, while strong inhibition would lead to suppression of kinase activity all along the axon, blocking axonal growth [43]. However, as recently suggested [31], a complete understanding of the role of GSK3β in axon growth and regeneration required additional experimentation using *in vivo* genetic models, as was performed in the current work. Here, we demonstrate that *in vivo* not only reduced GSK3β activity results in increased axon regeneration, but also, by using the cre^+^GSK3βlox/lox mice, we show that total ablation of GSK3β increases axonal regeneration, ruling out the need of a GSK3β gradient to enable axonal growth.

Given the wide array of GSK3β substrates, several studies have probed the downstream molecular mechanism by which GSK3β controls axonal growth. As referred to above, one of the working models was that GSK3β activity should be precisely controlled so that activity towards one subset of substrates would be specifically blocked, while activity towards others would be preserved [43]. In this model, inhibition of GSK3β activity towards CRMP-2 would allow its binding to microtubules promoting their polymerization, while maintenance of the activity towards MAP1B would maintain microtubules in a dynamic state, essential for axon growth. More recently, another model proposed CLASPs as the physiological target of GSK3 to exert control over axon growth [44]. In this model, promotion of axon growth occurred via moderate inhibition of GSK3 activity leading to the non-phosphorylation of some, but not all, GSK3 sites in CLASPs. This would allow CLASPs to specifically associate with microtubule plus ends promoting their stabilization [44]. Both models required partial inhibition of GSK3β to promote axonal growth and regeneration. However, we demonstrate that total GSK3β inhibition promotes axonal growth *in vivo* with a concomitant increase in microtubule growth speed in the growth cone. In this context, we show that the effects of GSK3β deletion are not mediated by decreased P-CLASP2 as in WT neurons, a non-phosphorylatable CLASP2 mutant leads to increased microtubule stabilization. Moreover, our results show that phosphorylation of MAP1B, although being sufficient to increase microtubule growth speed, is unnecessary for this effect. Interestingly, after SCI, increased levels of phosphorylated MAP1B have been reported in retraction bulbs of transected axons and in pre-apoptotic axotomized neuronal somata [45]. In our settings, overexpression of WT MAP1B did not influence microtubule growth speed. MAP1B was recently shown to interact with EB1/3 and to sequester these proteins [46]. Nevertheless, in that report, microtubule growth speed of MAP1B knockout hippocampal neurons was unaffected in the growth cone [46]. In DRG neurons from MAP1B knockout mice, whereas no differences were found in the overall neurite length, higher branching and impaired growth cone turning were present [47]. These data suggest that rather than playing a role in neurite growth, MAP1B is important for growth cone turning and branch formation during plastic changes in the adult.

More importantly, our results show that CRMP-2 is the primary downstream target of GSK3β that mediates the regulation of microtubule dynamics in the growth cone as demonstrated by reversion of increased microtubule growth speed of GSK3β-depleted neurons upon overexpression of a phospho-mimetic CRMP-2 mutant. Moreover, we demonstrate that the GSK3β-CRMP-2 pathway participates in the conditioning lesion effect, as overexpression of the phospho-mimetic T/D-CRMP-2 mutant partially reversed the increased microtubule growth speed of conditioned neurons leading to decreased axonal growth, and overexpression of the phospho-resistant T/A-CRMP-2 mutant attenuated the inhibitory effect of myelin in naïve DRG neurons. Besides being phosphorylated by GSK3β at Thr514 [39], CRMP-2 is also phosphorylated by Rho-associated protein kinase (ROCK) but at an alternative residue, Thr555 [48], downstream of both Myelin-associated glycoprotein (MAG) and Nogo-66 [49]. Interestingly, repulsive guidance molecule A (RGMa) inhibits axon growth by inducing CRMP-2 phosphorylation via both ROCK and GSK3β signaling [36]. However, in that study, details on which CRMP-2 phosphorylation (Thr514 or Thr555) was assessed are lacking, and GSK3β inhibition was performed in cultured neurons using a GSK3β antibody in the media and not a specific cell-permeable GSK3β inhibitor. Taken together, our data demonstrate that, irrespective of ROCK-mediated CRMP-2 phosphorylation at Thr555, inactivation of CRMP-2 phosphorylation at Thr514 by GSK3β is sufficient to reverse myelin inhibition in DRG neuron cultures. Our results point towards modulation of CRMP-2 activity as a therapeutic target to induce axonal regeneration. Further suggesting that CRMP-2 is a central target to design strategies to achieve release of myelin inhibition, overexpression of the phospho-resistant mutant T555A-CRMP-2 (the Rho-kinase phosphorylation site) counteracts the inhibitory effect of MAG on postnatal cerebellar neurons [49]. Additionally, protein phosphatase 2A promotes axonal growth by dephosphorylating CRMP-2 [50]. The involvement of CRMP-2 in several neurodegenerative diseases raised the need for developing therapeutic strategies targeting its functions. In this respect, drugs regulating CRMP-2 levels and activity are currently being tested in the settings of neurodegeneration [51].

## Conclusions

Our work shows that microtubule dynamics in the growth cone are higher in conditions of increased axonal growth, such as after conditioning lesion. This alteration is achieved following inactivation of the GSK3β-CRMP-2 pathway and is accompanied by enhanced axon regeneration through the glial scar. Based on the present results, studying the effect of CRMP-2-specific drugs, capable of modulating its activity, should be further developed in SCI and in other conditions where axonal regeneration needs to be promoted.

## Methods

### Animals

All animal experiments were performed according to national and European rules. The protocols described in this work have been approved by the IBMC Ethical Committee and by the Portuguese Veterinarian Board. GSK3β knockout heterozygous mice (GSK3β(+/-)) [17] were bred with C57/B6 mice and the resulting GSK3β(+/-) and WT littermates were selected as described [17]. Homozygous GSK3β Ser9Ala knockin (KI) mice, where codon encoding Ser9 of GSK3β was changed to encode the nonphosphorylatable Ala residue, were a kind gift from Dr Dario Alessi, Dundee University [16]. To generate experimental GSK3β Ser9Ala KI and the respective WT control mice, GSK3β Ser9Ala KIs were initially crossed with C57/B6 mice and the resulting GSK3β Ser9Ala heterozygous KI mice were inter-crossed such that GSK3β Ser9Ala KI and WT animals were generated and selected as described [16]. GSK3β floxed mice (GSK3βlox/lox) [22] and Slick-H mice (a kind gift from Dr Guoping Feng, Duke University Medical Center; mice that coexpress inducible-CreER^T2^ and YFP under the control of the Thy1 promoter [23]) were used to generate neuronal-specific conditional GSK3β knockout mice (cre^+^GSK3βlox/lox). For that, GSK3βlox/lox were crossed with Slick-H mice. Cre^+^GSK3βlox/wt mice were selected and then crossed to GSK3βlox/wt mice such that cre^+^GSK3βlox/lox and cre^+^GSK3βwt/wt mice were generated. Given the neuroprotective effects of tamoxifen [52,53], tamoxifen-treated cre^+^GSK3βlox/lox animals were compared to tamoxifen-treated cre^+^GSK3βwt/wt mice. In all experiments animals of either sex were used.

### Surgeries

For sciatic nerve injury, the sciatic nerve was transected at the mid thigh level. For spinal cord injury (SCI), laminectomy was performed at the T7 level in rats and at the T9/10 level in mice; dorsal hemisection or complete spinal cord transection were done using a micro feather ophthalmic scalpel. For conditioning lesion, animals were subjected to sciatic nerve transection and one week later to dorsal spinal cord hemisection.

### Phospho-site broad signaling pathway screen

DRGs (L4-L6) from a pool of six rats eight- to ten-weeks-old subjected to either SCI (dorsal hemisection) or conditioning lesion (CL) were sacrificed one week after SCI. DRG were homogenized in lysis buffer (20 mM 4-morpholinepropanesulfonic acid (MOPS), 2 mM ethylene glycol tetraacetic acid (EGTA), 5 mM ethylenediaminetetraacetic acid (EDTA), 30 mM NaF, 60 mM β-glycerophosphate, 20 mM sodium pyrophosphate, 1 mM sodium orthovanadate, 1% Triton X-100, 1% dithiothreitol (DTT), 1 mM phenylmethylsulfonyl fluoride (PMSF) and protease inhibitor cocktail (GE Healthcare, Carnaxide, Portugal)). Protein extracts (500 μg) were analyzed using the Kinexus phospho-site broad signaling pathway screen version 1.3 (KPSS-1.3, Kinexus Bioinformatics Corp, Vancouver, Canada). This screen examines 38 phosphorylation sites in 32 proteins with antibodies that recognize specific phosphorylated epitopes. The intensities of signals for target protein bands on the Kinetworks immunoblots were quantified as described [54]. Proteins with a fold change CL/SCI lower than 0.75 or higher than 1.25 were selected.

### Western blotting

DRGs (L4-L6) and the SCI site (3 mm rostral and 3 mm caudal to the lesion site) from either eight- to ten-week-old rats or WT and GSK3βSer9Ala KI mice with SCI or conditioning lesion were processed as described above. Lysates of neuronal cultures overexpressing GSK3β substrates (as described below) were prepared in phosphate-buffered saline (PBS) with 0.03% triton, 1 mM sodium orthovanadate and protease inhibitor cocktail. Protein lysates (25 to 50 μg/lane) were separated on either 12% or 3% to 8% gradient (in the case of MAP1B and CLASP2) SDS-PAGE gels and transferred to nitrocellulose (Amersham, Carnaxide, Portugal). Membranes were blocked and incubated overnight at 4°C in 5% bovine serum albumin (BSA) in tris-buffered saline with Tween (TBST) with the following primary antibodies: rabbit anti-P-AktSer473 (1:2000; Cell Signaling, Leiden, The Netherlands), rabbit anti-Akt (1:1000; Cell Signaling), rabbit anti-P-GSK3βSer9 (1:1000; Cell Signaling), rabbit anti-P-GSK3βTyr216 (1:2000; Santa Cruz Biotechnology, Heidelberg, Germany), mouse anti-GSK3α/β (1:3000; Santa Cruz Biotechnology), sheep anti-P-CRMP-2Thr509/514 (1:1000, Kinasource), sheep anti-CRMP-2 (1:500, Kinasource), rabbit anti-P-SrcTyr529 (1:500, Signalway antibody), rabbit anti-Fyn (1:1000, Cell Signaling), goat anti-Pyk2 (1:1000, Santa Cruz Biotechnology), rabbit anti-MAP1B (1:2500, kindly provided by Dr Itzhak Fischer, Drexel University College of Medicine, Philadelphia, PA, USA), rat anti-CLASP2 (kindly provided by Dr Helder Maiato, IBMC, Porto, Portugal), mouse anti-α-tubulin (1:5000, Sigma-Aldrich, Sintra, Portugal), mouse anti-β-actin (1:1000, Sigma-Aldrich) and mouse anti-glyceraldehyde 3-phosphate dehydrogenase (1:2000, Santa Cruz Biotechnology). Incubation with horseradish peroxidase-labeled secondary antibodies was performed for one hour at room temperature. Blots were developed using the enhanced chemiluminescence Western blot substrate (Pierce). Quantification was performed using the QuantityOne software (Bio-rad, Amadora, Portugal). For each experiment representative Western blots are shown. 

### GSK3β activity assays

A commercial kit (Sigma) was used for GSK3β activity assays. The samples analyzed were: the SCI site of eight- to ten-week-old rats with SCI or conditioning lesion, and the spinal cord (T8-T10) from eight-week-old GSK3βSer9Ala KI mice, GSK3β(+/-) mice or the respective WT controls. Briefly, 250 μg of protein lysate (n = 3 samples/condition) were incubated with the provided anti-GSK3β antibody in Protein G beads for three hours at 4°C. The kinase assay was performed in the immunoprecipitates using a GSK3β substrate and γ-^32^P-ATP. Control reactions without GSK3β antibody were performed. Results are presented as fold change relative to controls.

### Measurement of phosphatase activity

Phosphatase activity of the SCI site from eight- to ten-week-old rats with either SCI or conditioning lesion (n = 3 per condition) was assayed in 96-well microtiter plates using a tyrosine phosphatase assay system (Promega, Carnaxide, Portugal). Protein extracts (25 μg) were incubated with 100 μM of a P-GSK3βTyr216 peptide (Abcam, Cambridge, UK) at 30°C for 30 minutes. The released phosphate was determined by measuring the absorbance at 620 nm. Control reactions without the P-GSK3βTyr216 peptide were performed.

### Primary cultures of DRG neurons and neurite outgrowth assays

Primary cultures of DRG neurons were performed as described [55]. Briefly, all DRG (when analyzing naïve neurons) or L4-L6 DRG (when comparing naïve with conditioned neurons that is, neurons obtained from animals with sciatic nerve transection performed one week before DRG removal) from eight- to ten-week-old Wistar rats, or whenever indicated, from GSK3β genetic mouse models, were cultured for 12 hours. For transfection, the 4D Nucleofector Amaxa system (Lonza, Barcelona, Spain, CM#138 program) was used with 0.4 μg pmaxGFP™ (Lonza) or 0.4 μg pmaxGFP™ plus 2 μg pCMV5-Fyn Lys299Met (Fyn kinase-dead, a kind gift from Dr Marilyn Resh, Memorial Sloan-Kettering Cancer Center, New York, NY, USA). After transfection, cells were left in suspension for 24 hours and thereafter plated in poly L lysine (PLL)-laminin coated coverslips for 12 hours. For cultures with myelin, DRG neurons were isolated from mice at postnatal day 6 (P6). After transfection, DRG neurons were left in suspension for 24 hours and thereafter plated in PLL-laminin plus 3 μg myelin coated coverslips for 12 hours. Myelin was isolated from the spinal cord of 16-week-old WT mice as previously detailed [56]. To measure neurite outgrowth, immunocytochemistry for βIII-tubulin was done. The length of the longest neurite was traced with neuronJ [57]. The number of neurons traced was: for rat DRG neurons, 77 to 136; for GSK3Ser9AlaKI neurons, 29 to 100; for cre^+^GSK3βlox/lox neurons, 48 to 66 YFP + neurons.

### Overexpression of GSK3β substrates in neuronal cultures

Substrate residues phosphorylated by GSK3β were mutated to generate phospho-resistant or phospho-mimetic mutants. Mutations were generated by polymerase chain reaction (PCR)-based site-directed mutagenesis using QuickChange XL (Agilent Technologies, Santa Clara, CA, USA). For MAP1B, double mutants of the residues S1260 and T1265 were done to either Ala (ST/AA-MAP1B) or Asp (ST/DD-MAP1B) in pEGFP-C1 bearing full-length WT MAP1B. Neurons were co-transfected with EB3-mCherry and each of the MAP1B constructs, and microtubule growth speed was assessed. For CLASP2 adenoviruses, full-length WT or phosphomutant (9S/A-CLASP2 or 8S/D-CLASP2) EGFP-CLASP2α [26] was subcloned into pENTR/D-TOPO and recombined into pAd/CMV/V5-DEST using Invitrogen’s Gateway system, Carcavelos, Portugal. Adenovirus particles were generated and purified as previously described [27,58]. For CRMP-2, single amino acid mutations of the residue T514 to either Ala (T/A-CRMP-2) or Asp (T/D-CRMP-2) were generated, in a construct of full-length WT CRMP-2 (FLAG tagged at the N-terminus) cloned into the pRK5 expression vector, and co-transfections with EB3-mCherry or pEGFP-C1 were performed. For analysis of the relative expression of each mutant, the neuronal cell line CAD was used [59].

### Analysis of microtubule growth speed

DRG neurons from eight- to ten-week-old animals were nucleofected with either a truncated version of EB3-GFP (a construct containing aminoacids 1 to 200 of EB3, artificially dimerized by the addition of the leucine zipper domain of GCN4, cloned into the pEGFP-N1 vector, that efficiently accumulates at microtubule tips [60]) or EB3-mCherry, which contains full-length EB3 cloned into the mCherry-N2 vector (a kind gift from Dr Victor Small, Institute of Molecular Biotechnology, Vienna, Austria). After transfection, cells were left in suspension for 24 hours and then plated for 12 hours. For high resolution of EB3 comets, time-lapse recordings were performed for 100 frames every two seconds at 37°C on a Spinning Disk. Extended imaging periods required to correlate microtubule growth speed and axon growth were done by acquisition of 129 to 230 frames every five seconds at 37°C on a Spinning Disk. Kymographs were made using a Matlab script (LAPSO) [61]. The comet density (number of growing microtubules in a growth cone) was defined by counting the number of EB3 comets per frame per growth cone area. To quantify the distance of microtubule tips to the leading edge of the growth cone, a plot profile for EB3 fluorescence intensity versus distance was performed as previously described [62]. Briefly, EB3 fluorescence was measured from the tip of the growth cone up to 10 μm along the growth cone using ImageJ analysis software. A minimum of 61 microtubules from at least 11 neurons was quantified.

### Immunocytochemistry

DRG neurons from eight- to ten-week-old rats (either naïve or conditioned) and naïve neurons transfected with the Fyn kinase dead construct were fixed in either formalin or 2% paraformaldehyde (PFA) (in the case of transfected cells). Neurons from eight-week-old GSK3β(+/-) mice, cre^+^GSK3βlox/lox mice or the respective controls, were fixed with 2% PFA in cytoskeletal protection buffer (65 mM piperazine-N, N′-bis (2-ethanesulfonic acid) (PIPES), 25 mM 4-(2-hydroxyethyl)-1-piperazineethanesulfonic acid (HEPES), 10 mM EGTA, 3 mM MgCl_2_, 0,1% Triton X-100) for 15 minutes at room temperature. Cells were permeabilized with 0.2% triton X-100 (with the exception of cells fixed with the cytoskeletal protection buffer) and blocked with 5% normal donkey serum in PBS. Incubation with rabbit anti-P-GSK3βTyr216 (1:20; Santa Cruz Biotechnology), mouse anti-GSK3β (1:500; BD Transduction Laboratories, Oeiras, Portugal), rat anti-tyrosinated tubulin (1:100; Serotec, Oxford, UK), mouse anti-acetylated tubulin (1:1000; Sigma), sheep anti-P-CRMP-2Thr509/514 (1:200; Kinasourse), rabbit anti-P-MAP1BThr1265 (1:100, Novus Biologicals Cambridge, UK), and mouse anti-βIII-tubulin (1:2000; Promega) antibodies in blocking buffer was performed for one hour at room temperature. For calculating the intensity ratios for P-GSK3βTyr216/GSK3β, P-CRMP-2/βIII-tubulin and P-MAP1B/βIII-tubulin, the background of the photographs was subtracted and images were analyzed with the ImageJ plugin *Ratio Plus* generating an image where the mean value of the intensity ratio between the two channels in the growth cones was obtained. The ratio of acetylated versus tyrosinated α-tubulin was determined by measuring the fluorescence intensities of acetylated α-tubulin and of tyrosinated α-tubulin with ImageJ. Measurements were done in the entire growth cone area selected using the tyrosinated α-tubulin channel, after background subtraction for each channel. For the cre^+^GSK3βlox/lox mice, only YFP^+^ cells were analyzed. Data were analyzed with Prism5 (GraphPad) and the mean ± standard error of the mean (SEM) was calculated.

### Quantification of YFP-positive axons within the lesion area

In cre^+^GSK3βlox/lox mice, we quantified the number of YFP^+^ axons within the lesion area. The same images used for the quantification of dorsal column axons were used. Image analysis was performed using the Fiji software. After background subtraction, the threshold was automatically adjusted using the ‘Li’ algorithm [63]. Subsequently, the percentage of the lesion area occupied by the YFP signal was calculated.

### GSK3 immunohistochemistry

Paraffin sections of DRGs (L4 to L6) from eight-week-old cre^+^GSK3βlox/lox and cre^+^GSK3βwt/wt mice were blocked and incubated overnight at 4°C with the following antibodies: anti-GSK3β (1:100; Cell Signaling), anti-GSK3α (1:100; Cell Signaling), and anti-GFP (1:1000; Clontech, Lisboa, Portugal). Subsequently, incubation was performed with the respective fluorescent secondary antibodies and slides were mounted in vectashield with 4′,6-diamidino-2-phenylindole (DAPI.

### Analysis of regeneration of dorsal column fibers

Eight- to ten-week-old Wistar rats or GSK3β genetic mouse models were subjected to spinal cord dorsal hemisection and allowed to recover for five weeks. A group of animals was conditioned one week prior to SCI by transecting the left sciatic nerve. Four days prior to euthanasia, 2 μL of 1% CT-B (List Biologicals, Campbell, CA, USA) was injected in the left sciatic nerve. Animals were perfused with formalin, tissues were cryopreserved in sucrose and sectioned at 50 μm. Consecutive spinal cord sagittal sections were collected for free floating immunohistochemistry with anti-CT-B (1:30000; List Biologicals). Antigen detection was performed following incubation with biotinylated horse anti-goat (1:200; Vector, Peterborough, UK) either with extravidin peroxidase (1:1000; Sigma) or extravidin Alexa 568 (Invitrogen). Image analysis was done with the Photoshop CS5 (for optical microscopy) or the Fiji software (for confocal microscopy). Dorsal column fibers were quantified by counting the total number of axons within the glial scar in 1-in-3 sections. The length of the longest CT-B labeled axon found rostrally to the injury site, was measured using as the origin a vertical line placed at the rostral end of the dorsal column tract (that is, where CT-B labeling accumulates). Lesion margins were evident under phase-contrast optics as a distinct change in the appearance of the structure of the white and grey matter was observed. Total axon number/animal was calculated by multiplying the counted number by three.

### Analysis of regeneration of raphespinal serotonergic axons

Eight- to ten-week-old GSK3β(+/-) mice and WT littermates were subjected to complete spinal cord transection and allowed to recover for five weeks. Free floating immunohistochemistry with rabbit anti-5-HT (1:20000; Immunostar, Hudson, WI, USA) was performed. Images were taken on a confocal microscope (Leica) and analysis was done with Fiji. 5-HT + axons were quantified along the rostral-caudal axis by counting the total number of axons in a 3,000 μm area caudal to the injury site in eight to ten sections per animal. Cross-sections from uninjured spinal cords obtained at the T7 level were also processed for 5-HT immunostaining as above and 5-HT immunoreactivity was quantified in four sections/animal using FeatureJ software and normalized for the total area of the spinal cord. The glial scar area was evaluated using an anti-GFAP antibody (1:500, DAKO, Queluz, Portugal). The injury area, corresponding to the GFAP-negative area, was measured using Photoshop CS5.

### Behavioral assessment

Eight- to ten-week-old animals with complete transection of the spinal cord were assessed for locomotor recovery using the Basso mouse scale [64] at day one and weekly until five weeks after the injury. The observer was blinded to the animal’s genotype.

### Data analysis

Data are shown as mean ± SEM. Statistical significance was determined by Student’s t test or Tukey’s test (one-way analysis of variance (ANOVA)).

## Competing interests

The authors declare that they have no competing interests.

## Authors’ contributions

MMS coordinated the project. MAL and MMS conceived and designed experiments and wrote the manuscript. MAL, FMM, TES, HIP, AMM, MM, SV and VFS performed experiments. MAL, TES, HIP, SV and MMS analyzed data. HP, TW, CS and JRW provided unique reagents, contributed with valuable discussions and edited the manuscript. All authors read and approved the final manuscript.

## Supplementary Material

Additional file 1: Movie S1 Time-lapse live cell imaging of DRG neurons either naïve or conditioned (CL) transfected with GFP-EB3, and conditioned DRG neurons transfected with GFP-EB3 plus T/D-CRMP-2 (CL + T/D-CRMP2), taken using an Andor Revolution XD Spinning Disk confocal microscope. Scale bar: 5 μm. Total time: 200 seconds. Acquired at one frame per two seconds. 30x speed up. Click here for file

Additional file 2: Movie S2 Extended time-lapse live cell imaging of DRG neurons either naïve or conditioned (CL) transfected with GFP-EB3, and conditioned DRG neurons transfected with GFP-EB3 plus T/D-CRMP-2 (CL + T/D-CRMP2), taken using an Andor Revolution XD Spinning Disk confocal microscope. Scale bar: 5 μm. Total time: 770 seconds. Acquired at one frame per five seconds. 75x speed up. Click here for file

Additional file 3: Figure S1 Still frames from the extended time-lapse fluorescence microscopy of naïve and conditioned (CL) DRG neurons transfected with EB3-GFP, or conditioned neurons co-transfected with EB3-GFP and T/D-CRMP-2 (CL+T/D-CRMP-2). Scale bar: 5 μm. Click here for file

Additional file 4: Movie S3 Time-lapse live cell imaging of WT or GSK3β(+/-) DRG neurons transfected with GFP-EB3 taken using an Andor Revolution XD Spinning Disk confocal microscope. Scale bar: 5 μm. Total time: 200 seconds. Acquired at one frame per two seconds. 30x speed up. Click here for file

Additional file 5: Movie S4 Extended time-lapse live cell imaging of WT or GSK3β(+/-) DRG neurons transfected with GFP-EB3 taken using an Andor Revolution XD Spinning Disk confocal microscope. Scale bar: 5 μm. Total time: 645 seconds. Acquired at one frame per five seconds. 75x speed up. Click here for file

Additional file 6: Figure S2 Still frames from the extended time-lapse fluorescence microscopy of WT or GSK3β(+/-) DRG neurons transfected with GFP-EB3. Scale bar: 5 μm. Click here for file

Additional file 7: Movie S5 Time-lapse live cell imaging of cre^+^GSK3βwt/wt or cre^+^GSK3βlox/lox DRG neurons transfected with mCherry-EB3 (wt/wt or lox/lox, respectively), or cre^+^GSK3βlox/lox DRG neurons transfected with mCherry-EB3 plus T/D-CRMP-2 (lox/lox^+^T/D-CRMP2) taken using an Andor Revolution XD Spinning Disk confocal microscope. Scale bar: 5 μm. Total time: 200 seconds. Acquired at one frame per two seconds. 30x speed up. Click here for file

Additional file 8: Movie S6 Extended time-lapse live cell imaging of cre^+^GSK3βwt/wt or cre^+^GSK3βlox/lox DRG neurons transfected with mCherry-EB3 (wt/wt or lox/lox, respectively), or cre^+^GSK3βlox/lox DRG neurons transfected with mCherry-EB3 plus T/D-CRMP-2 (lox/lox^+^T/D-CRMP2) taken using an Andor Revolution XD Spinning Disk confocal microscope. Scale bar: 5 μm. Total time: 1,150 seconds. Acquired at one frame per five seconds. 75x speed up. Click here for file

Additional file 9: Figure S3 Still frames from the extended time-lapse fluorescence microscopy of cre^+^GSK3βwt/wt or cre^+^GSK3βlox/lox DRG neurons transfected with mCherry-EB3 (wt/wt or lox/lox, respectively), or cre^+^GSK3βlox/lox DRG neurons transfected with mCherry-EB3 plus T/D-CRMP-2 (lox/lox^+^T/D-CRMP2). Scale bar: 5 μm. Click here for file

Additional file 10: Movie S7 Time-lapse live cell imaging of rat WT naïve neurons expressing mCherry-EB3 alone (control) or with WT MAP1B, ST/AA-MAP1B or ST/DD-MAP1B, taken using an Andor Revolution XD Spinning Disk confocal microscope. Scale bar: 5 μm. Total time: 200 seconds. Acquired at one frame per two seconds. 30x speed up. Click here for file

Additional file 11: Movie S8 Time-lapse live cell imaging of rat WT naïve neurons expressing GFP-EB3 alone (control) or co-transfected with WT CRMP-2, T/A-CRMP-2 or T/D-CRMP-2, taken using an Andor Revolution XD Spinning Disk confocal microscope. Scale bar: 5 μm. Total time: 200 seconds. Acquired at one frame per two seconds. 30x speed up. Click here for file
