# Supplementary figures and images for: Neuronal deletion of GSK3β increases microtubule speed in the growth cone and enhances axon regeneration via CRMP-2 and independently of MAP1B and CLASP2
Source: BMC Biol. 2014 Jun 12;12:47. doi: 10.1186/1741-7007-12-47 (PMC4229956; doi:10.1186/1741-7007-12-47)

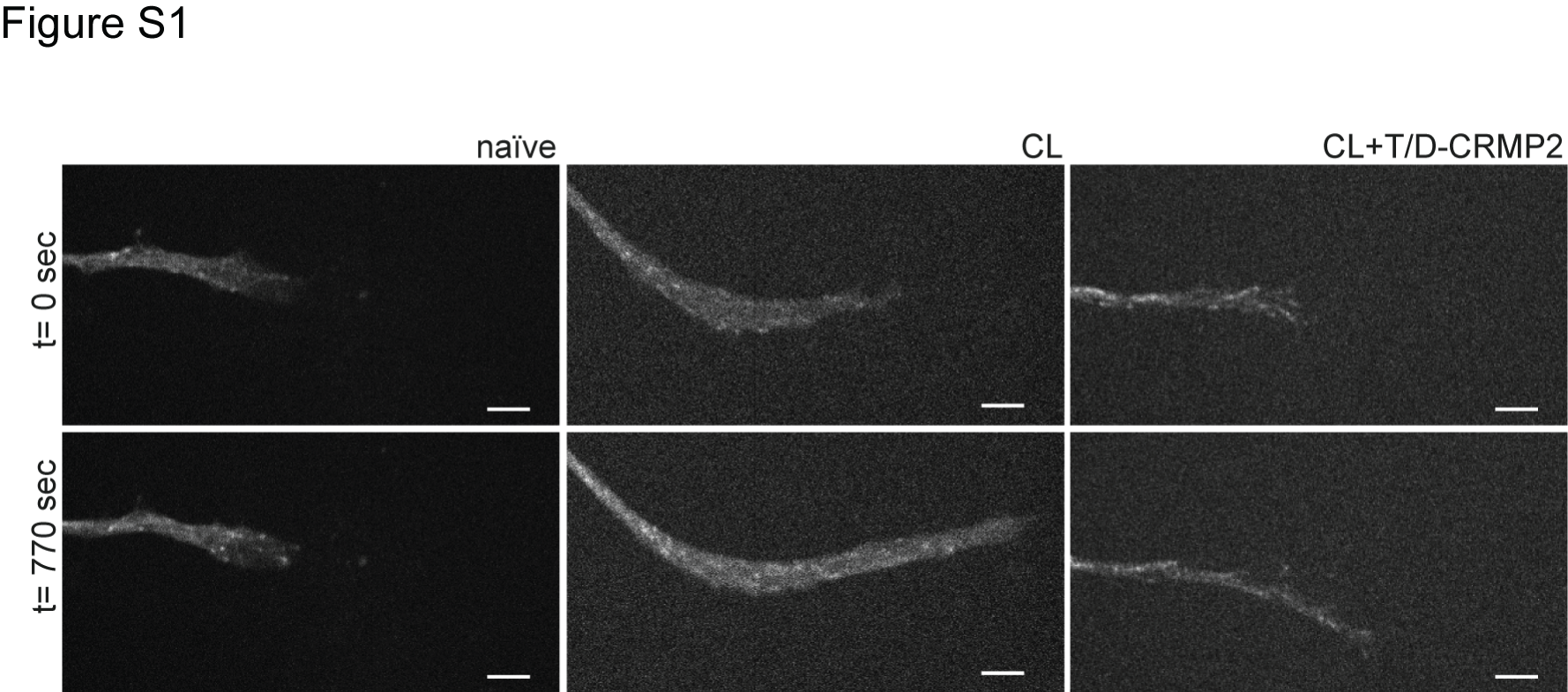

Supplement: Additional file 3: Figure S1 — Still frames from the extended time-lapse fluorescence microscopy of naïve and conditioned (CL) DRG neurons transfected with EB3-GFP, or conditioned neurons co-transfected with EB3-GFP and T/D-CRMP-2 (CL+T/D-CRMP-2). Scale bar: 5 μm. [file 1741-7007-12-47-S3.tiff]

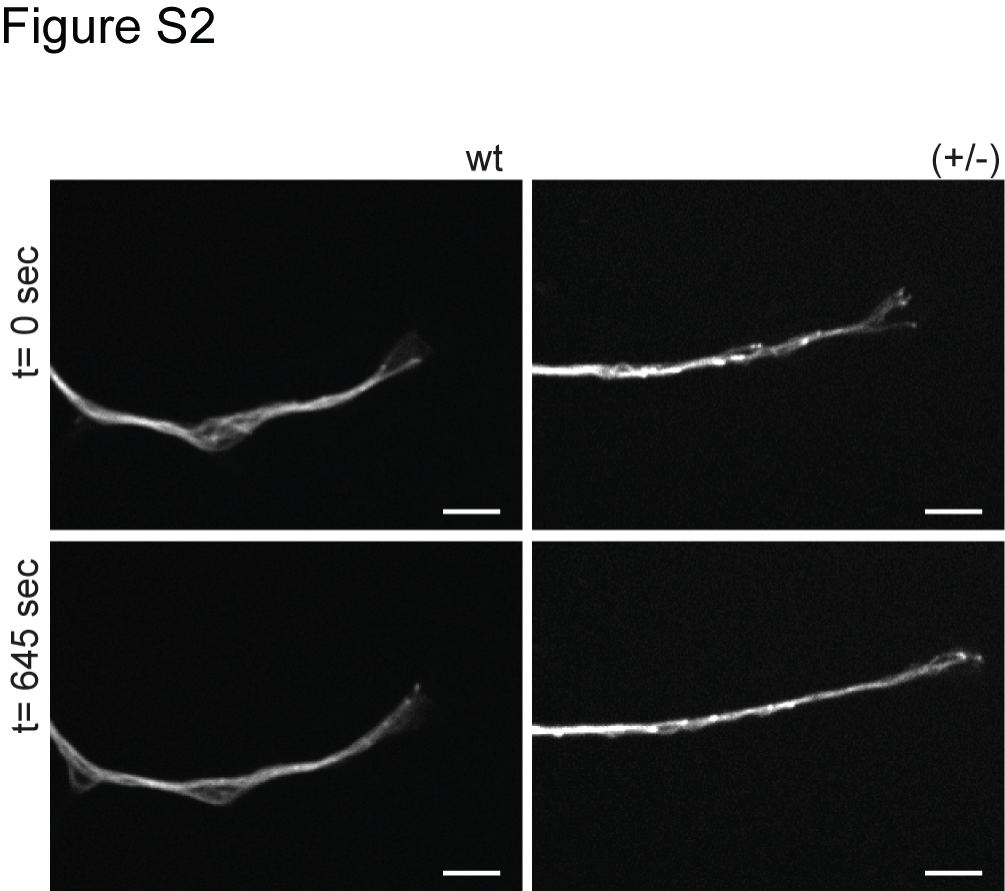

Supplement: Additional file 6: Figure S2 — Still frames from the extended time-lapse fluorescence microscopy of WT or GSK3β(+/-) DRG neurons transfected with GFP-EB3. Scale bar: 5 μm. [file 1741-7007-12-47-S6.tiff]

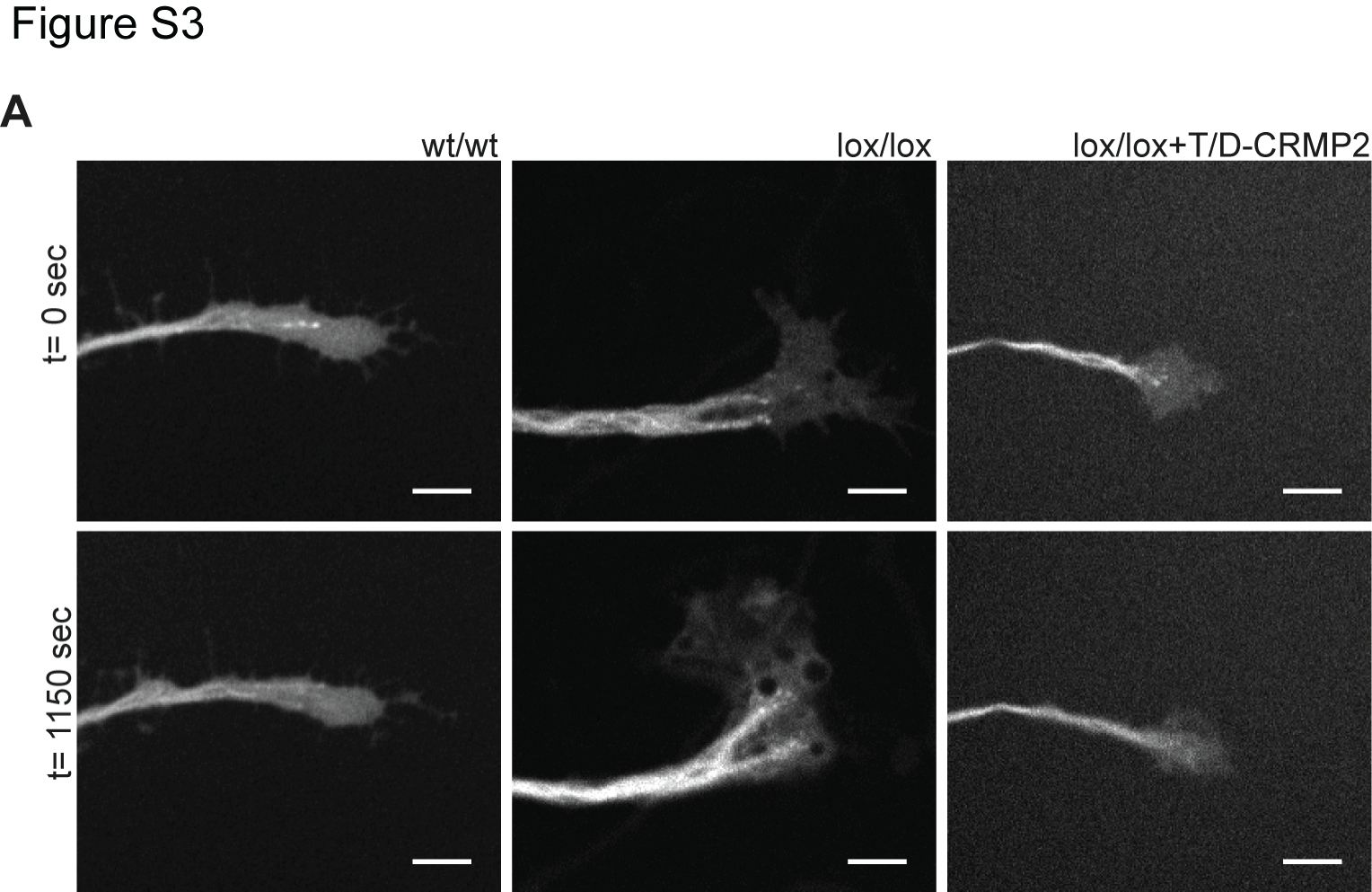

Supplement: Additional file 9: Figure S3 — Still frames from the extended time-lapse fluorescence microscopy of cre+GSK3βwt/wt or cre+GSK3βlox/lox DRG neurons transfected with mCherry-EB3 (wt/wt or lox/lox, respectively), or cre+GSK3βlox/lox DRG neurons transfected with mCherry-EB3 plus T/D-CRMP-2 (lox/lox+T/D-CRMP2). Scale bar: 5 μm. [file 1741-7007-12-47-S9.tiff]
